# Superconducting ternary hydrides: progress and challenges

**DOI:** 10.1093/nsr/nwad307

**Published:** 2023-12-07

**Authors:** Wendi Zhao, Xiaoli Huang, Zihan Zhang, Su Chen, Mingyang Du, Defang Duan, Tian Cui

**Affiliations:** Institute of High Pressure Physics, School of Physical Science and Technology, Ningbo University, Ningbo 315211, China; State Key Laboratory of Superhard Materials, College of Physics, Jilin University, Changchun 130012, China; State Key Laboratory of Superhard Materials, College of Physics, Jilin University, Changchun 130012, China; State Key Laboratory of Superhard Materials, College of Physics, Jilin University, Changchun 130012, China; State Key Laboratory of Superhard Materials, College of Physics, Jilin University, Changchun 130012, China; Institute of High Pressure Physics, School of Physical Science and Technology, Ningbo University, Ningbo 315211, China; State Key Laboratory of Superhard Materials, College of Physics, Jilin University, Changchun 130012, China; Institute of High Pressure Physics, School of Physical Science and Technology, Ningbo University, Ningbo 315211, China; State Key Laboratory of Superhard Materials, College of Physics, Jilin University, Changchun 130012, China

**Keywords:** high pressure, ternary hydrides, conventional superconductivity, electron–phonon coupling

## Abstract

Since the discovery of the high-temperature superconductors H_3_S and LaH_10_ under high pressure, compressed hydrides have received extensive attention as promising candidates for room-temperature superconductors. As a result of current high-pressure theoretical and experimental studies, it is now known that almost all the binary hydrides with a high superconducting transition temperature (*T*_c_) require extremely high pressure to remain stable, hindering any practical application. In order to further lower the stable pressure and improve superconductivity, researchers have started exploring ternary hydrides and had many achievements in recent years. Here, we discuss recent progress in ternary hydrides, aiming to deepen the understanding of the key factors regulating the structural stability and superconductivity of ternary hydrides, such as structural motifs, bonding features, electronic structures, electron–phonon coupling, etc. Furthermore, the current issues and challenges of superconducting ternary hydrides are presented, together with the prospects and opportunities for future research.

## Teaser

This work reviews the research progress of superconducting ternary hydrides, summarizes the key strategies targeted at improving superconductivity and lowering stable pressure, and presents current challenges and future prospects.

## INTRODUCTION

Superconducting materials have always been a focus of attention due to their unique quantum properties and great application prospects. Since Onnes discovered the superconductivity of mercury, the exploration of superconducting materials has lasted for more than a century. Many superconductors with higher superconducting transition temperature (*T*_c_) values have been gradually discovered, such as cuprate, iron-based, interface and organic superconductors, etc. The *T*_c_ values of cuprate superconductors can reach 133 K under ambient pressure [1], further increasing to 164 K at 31 GPa [2], which is the highest *T*_c_ on record. Based on the Bardeen–Cooper–Schrieffer (BCS) theory [3], metallic hydrogen is predicted to have room-temperature superconductivity [4,5] because the high-frequency vibration of hydrogen may be strongly coupling to electrons and lead to excellent conventional superconductivity. Meanwhile, the metallization of solid hydrogen requires extremely high pressure. Semi-metallic hydrogen has been observed at a pressure of >350 GPa and it remains a molecular solid at ≤440 GPa [6]. Researchers generally believe that metallic hydrogen requires an extremely high pressure of ∼500 GPa, which remains a long-standing challenge for experimentation. The breakthrough of this dilemma can be traced back to Ashcroft's proposal—that is, hydrogen-rich compounds can be stabilized at relatively low pressure by chemical precompression and exhibit high-temperature superconductivity [7]. From the existing hydrides in nature to the non-stoichiometric hydrides under high pressure, more and more binary hydrides with excellent superconductivity have been theoretically or experimentally found. Two exciting achievements in this field have been the discovery of covalent H_3_S [8–10] and clathrate LaH_10_ [11–16], whose superconducting transition temperatures are >200 K, marking a milestone in the history of superconductivity development. The discovery of H_3_S and LaH_10_ followed a completely different paradigm from all previous superconductors—that is, the fruitful symbiosis between theory, computation and experimentation. Numerous studies over the past few years have shown that this successful synergy will expand in the foreseeable future.

Thus far, the binary superconducting hydrides have been exhaustively investigated by using simulations [17], some of which have been confirmed by using experimental synthesis and characterization, such as ThH_9_, ThH_10_ [18], YH_6_ [19,20], BaH_12_ [21], CeH_9_ [22,23] and CaH_6_ [24]. Thanks to the ‘chemical precompression’ theory, most binary hydrides can remain stable at megabar pressures that are much lower than that required for the metallization of solid hydrogen. Nevertheless, these pressures are still too high for any practical application. Therefore, the next key issue or challenge is to lower the stable pressure of superconducting hydrides even to ambient pressure. Additionally, enhancing the superconducting transition temperature remains an ancient and important scientific issue. Notably, the critical temperature and pressure are equally crucial for optimizing the overall performance of superconducting hydrides, which means that a good balance between them should be maintained when further optimizing hydride superconductors.

In recent years, researchers have opened up new hunting ground in the search for superconducting hydrides with enhanced properties, typified by ternary hydrides with much higher freedom, which have richer chemical compositions and structural prototypes than binary systems, and thus may host even more novel properties. For example, the fluorite-type alloy backbone in LaBeH_8_ formed by the Be and H atoms therein is more easily stabilized at lower pressures than the pure hydrogen backbone, making LaBeH_8_ exhibit a high *T*_c_ of 185 K under moderate pressure [25]. Encouragingly, LaBeH_8_ has been successfully synthesized by relying on currently available experimental techniques [26]. Furthermore, recent experiments have found that ternary metal alloy hydrides exhibit enhanced stability or superconductivity compared with the corresponding binary parent structures, such as (La, Y)H_6_ [27], (La, Y)H_10_ [27], (La, Ce)H_9_ [28,29], (La, Al)H_10_ [30] and so on. On the other hand, element-doped binary hydrides can change the bonding of hydrogen atoms and increase the occupation of H-associated electronic states at the Fermi level, thus improving superconductivity and even obtaining room-temperature superconductivity. As an example, the introduction of Li atoms as charge doping in MgH_16_ rich in H_2_ molecular units effectively drives the dissociation of hydrogen molecules, forming Li_2_MgH_16_ with novel structural motifs, accompanied by *T*_c_ values of ≤473 K at 250 GPa [31]. Hence, multi-element collaborative regulation is an effective and promising method for optimizing high-temperature superconducting materials that provides feasible exploration ideas for the continued search for room-temperature superconductors or lowering the critical stability pressure.

Here, we review the research on ternary hydrides in recent years and discuss several important attempts to further lower the stable pressure and improve superconductivity. We summarize the general principles of high-temperature superconducting ternary hydrides. What are the key factors in regulating the superconductivity and stability of hydrides? What are the general characteristics of ternary hydrides with excellent superconducting properties? How can the stable pressure of hydrides be lowered and *T*_c_ enhanced? Finally, we provide an outlook on the challenges and opportunities for future research on superconducting ternary hydrides.

## LOWER THE SUPERCONDUCTING STABLE PRESSURE IN TERNARY HYDRIDES

In recent years, researchers have made many attempts to further reduce the superconducting stable pressure of ternary hydrides, reflected in the design of more complex structural motifs. For example, alloyed frameworks composed of some small-radius elements (e.g. Be, B, C, Si, etc.) with H atoms can be stabilized at lower pressures than pure hydrogen. Clathrate hydrides with appropriate combinations of metal elements as precompressors exhibit better stability than binary parent structures. The introduction of CH_4_ molecules into the typical H_3_S framework effectively lowers the structural stabilization pressure, etc. As such, we will introduce these successful attempts, aiming to deepen the understanding of the structure–property relationships of such intriguing superconducting materials.

### Precompressed H-based alloy backbone

**Figure 1.**
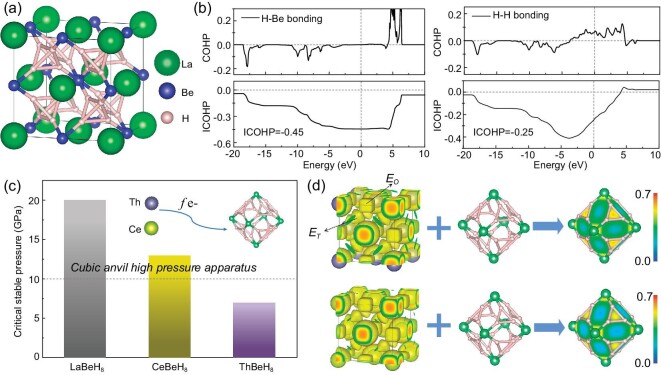
(a) Crystal structure of LaBeH_8_. (b) The calculated crystalline orbital hamiltonian population (COHP, top panel) and integrated crystalline orbital hamiltonian population (ICOHP, bottom panel) of H–Be bonds and H–H bonds of LaBeH_8_ at 100 GPa, adapted with permission from Ref. [25]. (c) The critical stable pressures of LaBeH_8_, CeBeH_8_ and ThBeH_8_. (d) The electron localization function (ELF) of the Th lattice in ThBeH_8_ (top panel) and the Ce lattice in CeBeH_8_ (bottom panel) overlaid on the fluorite-type cage at 100 GPa, respectively (isosurface = 0.4), adapted with permission from Ref. [32].

The ‘chemical precompression’ theory provides a strategy for the realization of high-temperature superconductivity via atomic hydrogen in hydrides at pressures that are much lower than those required for metallic hydrogen. Energy bands of atomic hydrogen in hydrides overlap with non-hydrogen elements, namely ‘precompressors’, suggesting that atomic hydrogen is predicted to be more stable than that in pure hydrogen metal. Because the overlapping of bands in superconducting hydrides are similar to those in alloys, these hydrides were called hydrogen-dominant metallic alloys. The ‘chemical precompression’ theory has been a great achievement in the design of hydride superconductors, as discussed above, but the pressures of all the designed hydrides are still >100 GPa and this limits their further application. Based on the description of the ‘chemical precompression’ theory, Zhang *et al.* proposed a ‘H-based alloy backbone’ theory to further lower the pressure of hydride superconductors [25]. The key idea of the ‘H-based alloy backbone’ theory is pre-compressing a binary H-rich system for a H-based alloy backbone with overlapping band structures to stabilized the atomic hydrogen. Regarding alloys, similar radii and electronegativity of elements promote the formation of uniform alloys, so the search for doped elements in H-based alloy backbones has mainly focused on Be, B, Al, Si and N elements. For example, element X (X = Sc, Ca, Y, Sr, La and Ba), with a large radius, precompresses the alloy lattice formed by the small-radius element Y (Y = Be, B, Al) and H to form a series of XYH_8_ compounds. As shown in Fig. 1a, Be and H atoms form a fluorite-type alloy backbone around the La atoms. This unique bonding environment allows the transfer of a large amount of charge to the H–H bond, inducing the generation of anti-bonding states at the Fermi level (see Fig 1b) and thus enhancing the electron–phonon coupling (EPC). Significantly, LaBeH_8_ is predicted to be thermodynamically stable at >98 GPa and can remain dynamically stable at 20 GPa with a high *T*_c_ (∼185 K). Very recently, LaBeH_8_ was successfully synthesized as the first crystallized template of superconducting ternary hydrides using precursors of equimolar La–Be alloys and NH_3_BH_3_ in a pressure range of 110–130 GPa, which is higher than the predicted thermodynamical pressure [26], suggesting that forward-looking simulations also could guide the discovery of complex stoichiometric hydride superconductors. Notably, critical temperature *T*_c_ values are increasing as the pressure decreases after the synthesis of LaBeH_8_ up to 110 K at 80 GPa. Diamond anvils for the synthesis of hydrides were always broken during depressurization as a result of hydrogen embrittlement and hydride LaBeH_8_ does not exhibit a superconducting dome as discovered in other high-temperature hydride superconductors such as H_3_S, LaH_10_ and CeH_9_. Therefore, if the diamond anvil is not broken during the decompression process, it is possible to maintain the superconductivity of LaBeH_8_ at <80 GPa because the dynamic stable critical pressure of LaBeH_8_ is significantly lower than its thermodynamic pressure. Note that the measured *T*_c_ of LaBeH_8_ is much lower than anticipated, but the crystal structure and volume agree with the prediction. The possible reasons are as follows. (i) Theoretically, different approaches lead to different results; the results from the McMillan–Allen–Dynes formula are lower than those from the Migdal–Eliashberg theory, and the estimated *T*_c_ values from the McMillan–Allen–Dynes formula for hydride LaBeH_8_ are close to the results obtained from the experiments by Song *et al.* (ii) Limited by the high-pressure experiments, the positions of both Be and H were not confirmed, suggesting that there may be a lot of defects in the sample of LaBeH_8_ in the diamond anvil cell (DAC). As a result, the *T*_c_ values of dissimilar samples are quite different from each other. To save the problem in novel ternary hydrides, the new approaches for superconductive predictions are worthy of being developed. With LaBeH_8_ as the parent structure, heavier elements Th and Ce are predicted to replace La atoms and produce stronger chemical precompression on the Be–H alloy lattice, thus forming more stable ThBeH_8_ and CeBeH_8_ (see Fig 1c) [32,33]. They are thermodynamically stable at 69 and 76 GPa, and dynamically stable at 7 and 13 GPa, corresponding to *T*_c_ values of 113 and 28 K, respectively. The *T*_c_ value of CeBeH_8_ is relatively low, mainly due to the suppression of superconductivity by *f* electrons. Furthermore, the stability of ThBeH_8_ and CeBeH_8_ have been revealed by the chemical template effect [34]. As shown in Fig. 1d, significant electron localization occurs at the octahedral E_O_ site and the tetrahedral E_T_ site in the metal (Th/Ce) sublattice interstitial region, indicating the occupation of local orbitals (quasi-atoms) here. The chemical template effect allows the quasi-atomic orbital electrons to be naturally doped into the BeH_8_ lattice, thereby minimizing the energy of the metal lattice and the BeH_8_ lattice, and ultimately providing a strong driving force for the stability of the metal and the BeH_8_ sublattice [34]. In addition, some hydrides isomorphic to LaBeH_8_, such as LaBH_8_ (156 K at 55 GPa), BaSiH_8_ (71 K at 3 GPa) and SrSiH_8_ (127 K at 27 GPa), are also predicted to be dynamically stable under moderate pressure, although their thermodynamic stable pressures are all >100 GPa [35–37]. Therefore, for LaBeH_8_ and its derived hydrides, the stability of atomic hydrogen is maintained in the form of a hydrogen sublattice alloying under lower pressure, thus hosting high-temperature superconductivity.

**Figure 2.**
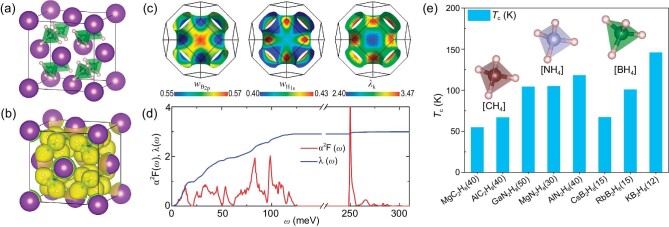
(a) Crystal structure of KB_2_H_8_. The BH_4_ units occupy the tetrahedral center of the face-centered cubic (fcc) potassium lattice. (b) The electron localization function of KB_2_H_8_ at 12 GPa. (c) Spectral weight on the Fermi surfaces for B-2*p* orbitals and H-1*s* orbitals and the strength of EPC *λ*_k_ on the Fermi surface, adapted with permission from Ref. [38]. (d) Eliashberg spectral function *α*^2^*F* (*ω*) and the electron–phonon integral *λ* (*ω*) for KB_2_H_8_ at 12 GPa, adapted with permission from Ref. [38]. (e) The *T*_c_ values of KB_2_H_8_ and its isomorphic hydrides at different pressures. The numbers in brackets represent the minimum dynamically stable pressure, in GPa.

Besides the ‘fluorite-like’ backbone experimentally confirmed in hydride LaBeH_8_, there are also hydrogen-rich molecular binary backbones precompressed by metal elements. The origin of the metallization of these hydride superconductors is metallic $\sigma $ bonds, which emerge in *sp*^3^-hydridized molecules in the backbone and exhibit excellent dynamic stability at low pressure, although most of them are of a metastable phase, suggesting that it could be difficult to synthesize these metastable phases. But these hydrides provide a possible route in metalizing binary molecular hydrides for high-*T*_c_ superconductivity. For example, inserting BH_4_ units (identifiable molecules) into the interstitial positions within using a fcc potassium sublattice can form KB_2_H_8_, in which each BH_4_ tetrahedron is also encapsulated by four K atoms [38] (see Fig. 2a). Therefore, this can also be seen as the precompression of the B–H alloy lattice by metal potassium atoms. The hydrogen-related *sp*^3^-hybridized *σ*-bonding states in KB_2_H_8_ are metalized and the weak covalent interaction exists between the nearest neighbor H atoms (see Fig. 2b), which increases the hydrogen-related electronic density of states (DOS) at the Fermi level. Remarkably, the mapping of the wave vector **k**-resolved EPC constant ${\lambda }_{{\bf k}}$ on the Fermi surface is similar to that of the spectral weights of the H 1s orbitals, revealing that the H 1s-related electronic states dominate the electron–phonon coupling (see Fig. 2c and d). KB_2_H_8_ is dynamically stable at 12 GPa and has a negative formation enthalpy relative to dissociation into elements. The further predicted *T*_c_ value can reach 146 K. Other alkali metals Rb and Cs can also form (Rb/Cs)B_2_H_8_ isomorphic to KB_2_H_8_, exhibiting high *T*_c_ of >100 K at 25 GPa [39] (see Fig. 2e). Furthermore, MC_2_H_8_ (M = Na, K, Mg, Al, Ga) formed by introducing CH_4_ molecules into the fcc metal lattice is also similar to the structure of KB_2_H_8_, which can exhibit high-temperature superconductivity under dynamic stable pressure of <100 GPa. In particular, the *T*_c_ of MgC_2_H_8_ can be increased from 13.4 K at 20 GPa to 55 K at 40 GPa, and the *T*_c_ of AlC_2_H_8_ can reach 67 K at 80 GPa [40]. Recently, AlN_2_H_8_ (118 K at 40 GPa), MgN_2_H_8_ (105 K at 30 GPa) and GaN_2_H_8_ (104 K at 50 GPa) were formed by inserting ammonium ions into the fcc metal lattice and have also been predicted to be stable under moderate pressure [41]. Although N elements with high electronegativity attract 1s electrons from H to fill the N-2*p* orbitals in the [NH_4_] unit, metal atoms (Ag, Mg, Ga, etc.) with lower electronegativity donate electrons to H, which promotes the dissociation of the N–H bond as well as the metallization of the hydrogen.

Very recently, Dias *et al.* reported that room-temperature superconductivity of *T*_c_ up to 294 K was measured in nitrogen-doped lutetium hydrides at a much lower pressure of 10 kbar [42]. If this result can be repeated, it will be quite exciting and important. Unfortunately, due to the difficulty of identifying light elements such as hydrogen and nitrogen by using x-ray diffraction, the composition and structure of this room-temperature superconducting phase, especially the actual stoichiometry and atomic position of hydrogen and nitrogen, cannot be fully resolved. Notably, this surprising room-temperature superconductivity has not been found in binary lutetium hydrides and recently reported Lu–N–H systems [43–49].

### Precompressed H_3_S-based framework

**Figure 3.**
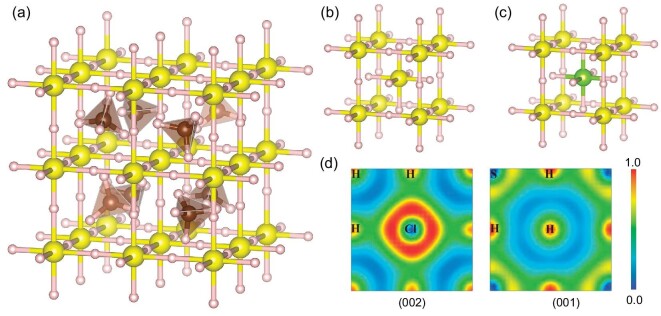
Several high-pressure covalent structures. (a) *I-*43*m* CSH_7_, adapted with permission from Ref. [50]. (b) *Im*-3*m* H_3_S. (c) *Pm*-3*m* H_6_SCl. (d) Calculated ELF on (002) and (001) planes for H_6_SCl at 90 GPa, adapted with permission from Ref. [51].

Covalent hydrides mainly rely on the covalent interaction between non-metallic elements and atomic hydrogen to maintain structural stability and exhibit covalent metallicity. The theoretically predicted covalent hydride H_3_S has been experimentally confirmed to have a *T*_c_ value of ≤203 K at 155 GPa [8–10,52,53]. It is crystallized in a cubic structure with two body-centered cubic [H_3_S] sublattices nesting each other. Its superior superconductivity is mainly attributed to the strong EPC caused by its covalent metallicity and the high-frequency phonon vibration modes driven by the lightweight H atoms. The discovery of H_3_S has greatly encouraged the superconducting community, and especially attracted the attention of researchers towards traditional superconductors. In 2020, Snider *et al.* measured room-temperature superconductivity of ≤288 K in carbonaceous sulfur hydrides at 267 GPa, but there are still many open questions surrounding this system, including the detailed composition and structure of the superconducting C–S–H system. Although this work has been withdrawn, it has sparked a series of research work on C–S–H under high pressure. Prior to this study, two independent theoretical works on the C–S–H system identified a metastable CSH_7_ that can maintain dynamic stability at 100 GPa [50,54], where the pressure is much lower than that of H_3_S. CSH_7_ can be seen as a hydride perovskite structure formed by the intercalation of methane into an H_3_S framework (see Fig. 3a). The ionic interactions between the CH_4_ guest molecules and the [SH_3_] host lattice enhance the chemical precompression, thereby reducing the stabilization pressure, but also suppresses superconductivity. Subsequently, researchers have theoretically studied C-doped H_3_S or performed comprehensive theoretical predictions on the phase diagram of the C–S–H system, and they did not find any thermodynamically stable ternary compounds. Some dynamically stable ternary compounds exhibit high-temperature superconductivity, such as CS_2_H_10_ (95 K at 50 GPa) [55], C_2_S_2_H_4_ (16.47 K at 300 GPa) [56], CSH_3_ (98 K at 250 GPa) [57], CS_3_H_13_ (142 K at 270 GPa) [58] H_3_S_0.917_C_0.083_ (189 K at 300 GPa) [59], etc., but their *T*_c_ values are still far from room temperature.

Recently, H_3_S has been predicted to be stable at lower pressures after replacing some S atoms with halogen elements (Cl and Br). H_6_SCl and H_6_SBr exhibit high *T*_c_ values of 155.4 and 136.4 K at 90 and 140 GPa, respectively [51]. The electronegativity of the Cl element is stronger than that of S, resulting in a stronger covalent bond between Cl and H than between S and H, thus keeping H_6_SCl stable at lower pressure (see Fig. 3c and d). PSH_6_ is also predicted to have a *T*_c_ value of 146 K at 130 GPa [60]. Generally, H_3_S-based covalent hydrides exhibit superconductivity close to that of H_3_S. However, whether they can achieve room-temperature superconductivity is still controversial.

### Enhanced stability by alloying clathrate hydrides

The clathrate hydrides are characterized by atomic-like hydrogen sublattices that encapsulate electropositive metal atoms, typically alkaline or rare earth (RE) and some actinide metals. The atomic radius, electronegativities and valence electrons of metal atoms play an important role in tuning the superconductivity and stability of hydrides. Multinary alloys constructed by mixing appropriate metal elements are expected to form ternary hydrides with more superior properties; especially RE metals have similar electronegativities and atomic radii, permitting their associated disordered solid solution alloys to be formed easily and stabilized under moderate pressures.

### *f*-shell metals enhanced chemical precompression

**Figure 4.**
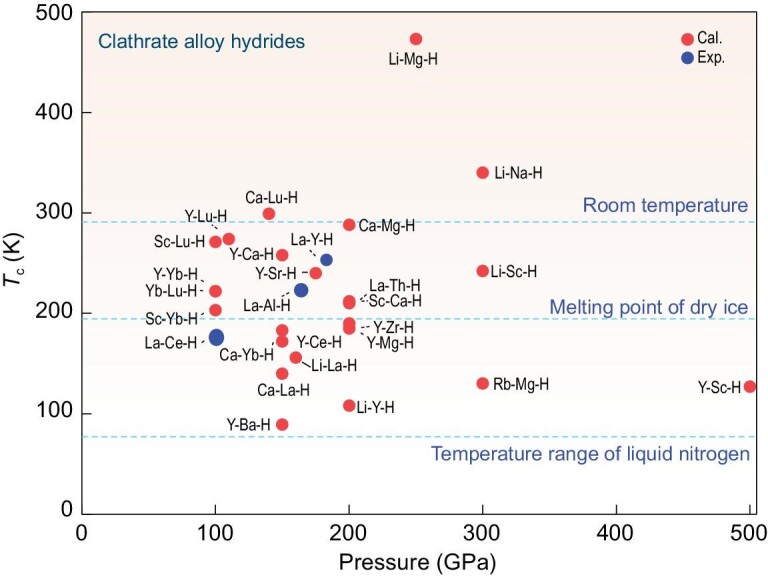
Some reported ternary clathrate alloy hydrides with *T*_c_ values above liquid nitrogen temperature. The red and blue dots represent the theoretical and experimental results, respectively. The *T*_c_ value is selected from the optimal value of each system.

**Figure 5.**
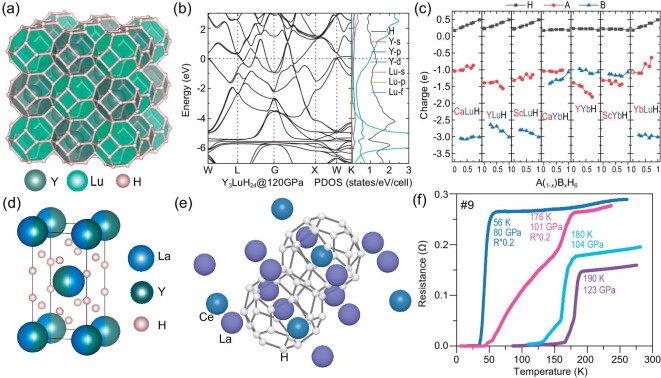
(a) Crystal structure of *Fm*-3*m* Y_3_LuH_24_. (b) Electronic band structures and projected density of states (PDOS) of *Fm*-3*m* Y_3_LuH_24_ at 120 GPa. (c) Charges transferred as a function of doping concentration between different elements in A_(1__–_*_x_*_)_B*_x_*H_6_. The positive and negative charge values of different elements represent the gain and loss of electrons, respectively, reproduced with permission from Ref. [63]. (d) The structure model of *I*4/*mmm*-(La, Y)H_4_, adapted with permission from Ref. [64]. (e) *P*6_3_/*mmc* (La, Ce)H_9__–1__0_. (f) The temperature dependence of the electrical resistance for the La–Ce–H sample in DACs #9, reproduced with permission from Ref. [28].

Systematic studies indicate that some heavy RE elements (e.g. Ce, Yb, Lu, etc.) occupying *f*-subshell electrons are considered great chemical ‘precompressors’ and their related hydrides are usually stable under lower pressures. As mentioned above, CeBeH_8_ can be stable under much lower pressure than LaBeH_8_ [33]. However, most of the heavier lanthanide hydrides are not considered promising for hosting high-temperature superconductivity due to the suppressive influence of *f* electrons on superconductivity and the maximum *T*_c_ value decreases rapidly once past La. This has been reflected in the binary systems of CeH_9_ [23], PrH_9_ [61] and NdH_9_ [62]. With the *f* electrons increasing, the superconductivity of these hydrides is gradually suppressed. To improve the superconductivity of these *f*-electron-containing metal hydrides, substitutional alloying has proven to be a promising approach. In recent years, many clathrate alloy hydrides have been predicted as high-temperature superconductors (see Fig. 4). Their superconducting properties are usually close to those of the binary parent structure. Especially, the theoretically predicted dynamic stability critical pressures of Y_3_LuH_24_ (283 K, 120 GPa, see Fig. 5a) and YLu_3_H_24_ (288 K, 110 GPa) are significantly lower than those of room-temperature superconductors YH_10_ and CaBeH_8_ without *f* electrons [63]. For the Lu-containing structures, the extra electrons in the 5*d* orbitals lead to the 4*f* moving away from the Fermi surface (see Fig. 5b), which reduces the negative effect of the *f* orbital electrons on the superconductivity. At the same time, the Lu element can donate more electrons to hydrogen than other metals (see Fig. 5c). The electrons obtained by H atoms increase with an increase in the Lu-doping concentration. The extra electrons weaken the H–H covalent bond, modulate the H–H bond length to influence the vibration of the hydrogen sublattice and ultimately enhance the EPC. On the other hand, note that the properties of the *f*-electron-containing metals are demonstrated with the accuracy achieved by the current computational capabilities. In practice, it is difficult to very accurately assess the effects induced by *f* electrons within the framework of density functional theory.

### Disordered alloy hydrides

Alloy hydrides have unique advantages in enhancing structural stability—especially, in many cases, in binary subunits that cannot exist independently can be stabilized in the form of solid solution crystallization. Semenok *et al.* synthesized a series of lanthanum–yttrium ternary hydrides in the pressure range of 170–196 GPa via the laser heating of La–Y alloys using ammonia borane, including (La, Y)H_6_ with an H_24_ cage [27], indicating that the unstable LaH_6_ is stable in this unique solid solution alloy. Furthermore, a disordered alloy tetrahydride *I*4/*mmm*-(La, Y)H_4_ with a high *T*_c_ of >90 K was experimentally synthesized at ∼110 GPa (see Fig. 5d) [64]. The La and Y atoms in *I*4/*mmm*-(La, Y) H_4_ occupy the same metal site with an equal probability of ∼50%. Importantly, compared with YH_4_, (La, Y) H_4_ can remain stable at lower pressures and is recoverable down to ∼80 GPa. Recently, the synthesis of ternary La–Ce–H compounds was first reported with a record *T*_c_ of ∼176 K at 100 GPa [28] (see Fig. 5e and f). The extrapolation of the upper critical magnetic field gives *H*_c2_(0) = 235 T at 100 GPa. Strikingly, the La–Ce–H system hosts higher *T*_c_ values at <130 GPa compared with the binary La–H and Ce–H phases, and the disordered state in the La–Ce–H system is considered to contribute to the significant enhancement of superconductivity and stability. At the same time, *P*6_3_/*mmc* (La, Ce)H_9_ with equal metal-atom occupancy was synthesized by using a La–Ce alloy with an initial ratio of 1:1 and ammonia borane (NH_3_BH_3_) as starting materials [29]. The composition of (La, Ce)H_9_ can be seen as La randomly replaces half of the Ce in CeH_9_ and occupies the same metal position as the Ce atom. This phase exhibits a high *T*_c_ of 148 K at 97 GPa that was enhanced to 178 K at 172 GPa. The big differences in *T*_c_ under the same pressure should come from the different ratios of the La–Ce alloy, indicating the important impact of the original alloys. In short, the experimentally reported La–Ce–H system provides new examples for the synthesis of alloy hydrides, which not only show that substitutional alloying is a promising method to adjust and improve the comprehensive properties of superconducting hydrides, but also can be extended to more ternary or multicomponent high-entropy alloys. This will enhance confidence in the optimal superconducting hydrides by carefully screening the metal-element combinations of substitutional alloys.

Furthermore, the ternary alloy hydrides could be formed by the two non-hydrogen elements with larger differences in radius and electronegativity, although this is a great challenge. Recently, a new type of La–Al hydride was successfully synthesized via laser heating of La–Al alloy with ammonia borane in the pressure range of 146–183 GPa [30]. Importantly, by introducing Al, the predicted metastable *P*6_3_/*mmc* LaH_10_ phase becomes stable at 146 GPa showing superconductivity with a *T*_c_ of ∼178 K, and this value is enhanced to a maximum *T*_c_ of ∼223 K at 164 GPa. Inspired by the high *T*_c_ in these ternary hydrides, it is foreseeable that multicomponent alloy hydrides are still promising competitors for obtaining high-temperature or even room-temperature superconductivity in the future exploration of superconducting ternary hydrides.

## IMPROVE THE SUPERCONDUCTING TRANSITION TEMPERATURE IN TERNARY HYDRIDES

Within the BCS theory [3], the superconducting transition temperature *T*_c_ can be obtained via:


(1) \begin{eqnarray*}{k}_B{T}_c = 1.14\hbar \omega\exp\left[ {\frac{{ - 1}}{{N\left( 0 \right)V}}} \right]\end{eqnarray*}

where $\omega $ represents the average phonon frequency, $N( 0 )$ is the DOS at the Fermi level and *V* is the pairing potential between two electrons resulting from the electron–phonon interaction. The lattice vibration of hydrogen can drive high phonon frequencies, suggesting that hydrogen-rich compounds were predicted to be potential high-temperature superconductors. Allen and Dynes further proposed the widely used semi-empirical formula for estimating *T*_c_ [65]:


(2) \begin{eqnarray*}{T}_c = {\omega }_{\rm log}\frac{{{f}_1{f}_2}}{{1.2}}\exp \left( {\frac{{ - 1.04\left( {1 + \lambda } \right)}}{{\lambda - {\mu }^* - 0.62\lambda {\mu }^*}}} \right)\end{eqnarray*}

where ${f}_1$ and ${f}_2$ are two correction factors and ${\mu }^*$ represents the Coulomb pseudopotential. The logarithmic average phonon frequency ${\omega }_{\rm log}\ $and EPC parameter $\lambda $ are given by:


(3) \begin{eqnarray*}{\omega }_{\rm log} = {\mathrm{exp}}\left( {\frac{2}{\lambda }\int \frac{{{\rm d}\omega }}{\omega }{\alpha }^2F\left( \omega \right)\ln \left( \omega \right)} \right)\end{eqnarray*}

(4) \begin{eqnarray*}\lambda = 2\int \frac{{{a}^2F\left( \omega \right)}}{\omega }{\rm d}\omega. \end{eqnarray*}

Obviously, larger $\lambda $ and ${\omega }_{\rm log}$ are beneficial for obtaining high *T*_c_ for conventional superconductors, as well as for hydrides. It should be recognized, however, that, within a given structure, λ usually increases by lowering frequencies, which may lead to lattice instability. Differently from binary hydrides, the combination of different metal elements as precompressors causes the diversity of ground-state crystal structures and properties. Ternary systems with high-temperature or even room-temperature superconductivity can often coordinate the advantages of different elements to produce key features conducive to superconductivity, such as high symmetry of the structure, high H-derived DOS at the Fermi level and strong EPC. Next, we will introduce several important attempts to enhance superconductivity in ternary hydrides and summarize the key factors that trigger high-temperature and even room-temperature superconductivity.

### Maintain full atomization of hydrogen sublattice

**Figure 6.**
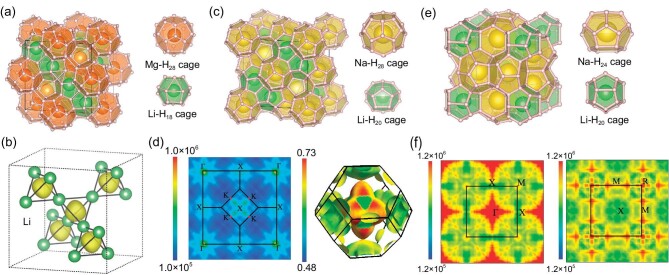
(a) and (b) The high-pressure structure of *Fd*-3*m* Li_2_MgH_16_ and the anionic electrons in the Li sublattice interstitial region therein, adapted with permission from Ref. [68]. (c) and (d) The high-pressure structure of Li_2_NaH_17_ and its 2D Fermi surface nesting function and 3D Fermi surface sheets in the Brillouin zone. (e) and (f) The high-pressure structure of LiNa_3_H_23_ and its 2D Fermi surface nesting in the Brillouin zone, adapted with permission from Ref. [70].

It is well known that the structural motifs of ternary hydrides are complex and are mainly affected by the synergistic effects of multiple factors, such as stoichiometry, hydrogen content, electronegativity, etc. Interestingly, ternary hydrides with atomic-like hydrogen lattices always stand out in the hunting ground of superconducting ternary hydrides, which is attributed to the tendency of atomic-like hydrogen sublattices to exhibit higher H-derived DOS at the Fermi level and stronger electron–phonon coupling. Generally, superhydrides containing more atomic hydrogen will have more excellent superconductivity. For example, YH_6_ [19,20,66], YH_9_ [20,67] and YH_10_ [12] in the binary Y–H system all have atomic-like hydrogen sublattices and their *T*_c_ values gradually increase. Hence, a strategy for improving the superconductivity of hydrides is to donate electrons in hydrogen-rich compounds with higher hydrogen content via metal doping to obtain atomic-like hydrogen. For example, metal atoms Li and Mg as electron donors provide abundant electrons for H_2_ molecular units in the parent structure *P*-1 MgH_16_, effectively driving the molecular dissociation and further expanding into an atomic clathrate hydrogen covalent lattice in metastable *Fd*-3*m* Li_2_MgH_16_ [31]. As shown in Fig. 6a, the H atoms in Li_2_MgH_16_ form H_18_ and H_28_ cages around the Li and Mg atoms, respectively. Notably, a Li atom under high pressure is reported to be an electride because pressure could drive the anionic electrons to accumulate outside the Li atom [68] (see Fig. 6b). These electrons are easily trapped in the H_18_ and H_28_ cages, resulting in the stabilization of the H cage and the enhancement of the DOS derived from H at the Fermi level. Therefore, metal Li doping induces strong electron–phonon coupling of Li_2_MgH_16_ and makes it exhibit a *T*_c_ value of ≤473 K at 250 GPa. Furthermore, room-temperature superconductivity was also found in the two metastable Li_2_ScH_16_ (281 K, 230 GPa) and Li_2_YH_16_ (285 K, 170 GPa), which are isomorphic to Li_2_MgH_16_ [69]. Unfortunately, Li_2_MgH_16_, Li_2_ScH_16_ and Li_2_YH_16_ are thermodynamically metastable under high pressure, which means that they are difficult to synthesize. Recently, two thermodynamically stable LiNa_3_H_23_ (310 K, 350 GPa) and Li_2_NaH_17_ (340 K, 300 GPa) have been predicted to have room-temperature superconductivity [70], in which hydrogen atoms surround Li and Na atoms to form different types of atomic clathrate hydrogen sublattices (see Fig. 6c–f). Their structural motifs are equivalent to the identified type I and II silicon clathrate geometry. The dominant H-derived DOS at the Fermi level and the strong Fermi surface nesting promote the generation of room-temperature superconductivity. Notably, the binary systems Ba_4_H_23_ [71], La_4_H_23_ [72] and Lu_4_H_23_ [43] with the same hydrogen motif as LiNa_3_H_23_ have been synthesized, but their superconductivity is not significant, which reveals the advantage of ternary hydrides in synergy with different element properties to enhance superconductivity. The room-temperature superconducting ternary hydrides discussed above fully reveal that keeping more hydrogen stable in the form of atomic-like states to fully excite the dominant advantage of hydrogen on DOS at the Fermi level is crucial to promoting *T*_c_ values.

### *s–d* boundary metal ion-doped hydrides

**Figure 7.**
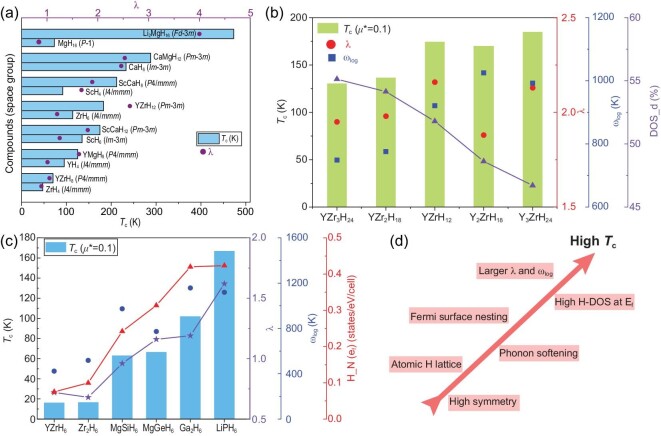
(a) Comparison of superconducting parameters of several binary hydrides and ternary systems obtained after the introduction of *s–d* boundary metals. (b) The *T*_c_ values of hexahydrides Y_3_ZrH_24_, Y_2_ZrH_18_, YZrH_12_, YZr_2_H_18_ and YZr_3_H_24_ at 200 GPa. DOS_d represents the proportion of *d* electronic states in the total DOS at the Fermi level. (c) The *T*_c_ values and related parameters of several A15-type hydrides, reproduced with permission from Ref. [77]. (d) Several key factors that are conducive to promoting superconductivity in hydrides.

Numerous studies on binary superconducting hydrides have shown that binary systems with excellent superconductivity tend to be distributed in the *s–d* boundary region of the periodic table (such as CaH_6_ [24,73], YH_6_ [19,20,66], LaH_10_ [11–16], etc.). Miao *et al.* believe that the metals near the *s–d* border can produce a stronger ‘chemical template effect’ than metals in other regions under pressure, which effectively assists the dissociation of H_2_ molecules and stabilizes the aromatic building units of the H sublattices [34]. Therefore, further doping of hydrides with active metals near the *s–d* border helps to enhance the template effect and ultimately improve the stability and superconductivity of the structure. For example, CaMgH_12_ obtained by replacing the general Ca atoms in CaH_6_ with Mg atoms has a lower formation enthalpy than the parent structure, showing enhanced superconductivity, and the predicted *T*_c_ value can be increased to 288 K [74] (see Fig. 7a). Similarly, ScCaH_8_ [75], ScCaH_12_ [75], YMgH_8_ [76], YZrH_12_ and YZrH_8_ [77], which can be obtained by replacing half of the metal atoms in ScH_4_, ZrH_6_, ScH_6_, YH_4_ and ZrH_4_, respectively, exhibit higher electron–phonon coupling than the parent structure, accompanied by higher *T*_c_ values. Semenok calls the *s–d* boundary region of the Mendeleev's Table a ‘lability belt’ [78], which is attributed to the fact that these elements are electronically labile under pressure—that is, their orbital populations are sensitive to the atomic environment, thereby inducing strong EPC. Indeed, the *s–d* boundary elements have low electronegativity, readily donating electrons to the H sublattice(s), breaking the strong H_2_ molecular bonds and promoting the production of metallic ground states. Furthermore, the filling of the outer electron shells of the element closely affects the superconductivity. Empirical rules suggest that excessive *d* and *f* electrons may inhibit superconductivity. Transition metals are usually rich in *d* electrons. The transition metal hydrides Li_5_MoH_11_ [79] and BaReH_9_ [80] synthesized in earlier experiments failed to allow the light H elements to dominate the superconductivity, so their *T*_c_ values at >100 GPa are <10 K. In turn, properly adjusting the excess *d* or *f* electrons near the Fermi level will help improve superconductivity. For example, several metastable hexahydrides in the Y–Zr–H system have been predicted to have high-temperature superconductivity (see Fig. 7b): Y_3_ZrH_24_, Y_2_ZrH_18_, YZrH_12_, YZr_2_H_18_, YZr_3_H_24_ [77]. The *T*_c_ value of Y_3_ZrH_24_ (185 K at 200 GPa) is significantly higher than that of YZr_3_H_24_ (131 K at 200 GPa), which is due to the fact that Zr contains more *d* electrons than Y atoms, and they tend to be localized at the Fermi level, thus suppressing the H-dominated superconductivity. Therefore, the increase in the Y atom ratio reduces the proportion of *d* electrons at the Fermi level, thereby enhancing the superconductivity. Similarly, in A15 hydrides, the *T*_c_ values of YZrH_6_ (16 K at 1 atm) and ZrH_3_ [81] (16.6 K at 10 GPa) with DOS dominated by *d* electrons are much lower than that of LiPH_6_ (167 K at 200 GPa) (see Fig. 7c). More importantly, LiPH_6_ has higher H-derived DOS than other A15 structures, inducing larger $\lambda $ and ${\omega }_{\rm log}$, thus exhibiting the highest value of *T*_c_ in A15 hydrides [82], and it is also superior to the superconductivity of P–H binary hydrides [83]. In short, even for hydrides with the same hydrogen structure motif, the high-temperature superconductivity always depends on the chemical composition that can drive high H-dominated DOS at the Fermi level, and hydrides doped with *s–d* boundary active metals tend to have such characteristics (see Fig. 7d). Another point that merits a mention is that doping binary hydride systems may also induce softening of phonon modes, and the softened vibration modes are more pronounced at the edge of dynamic stability, which is beneficial for improving electron–phonon coupling [84].

## CHALLENGE IN THEORETICAL CALCULATIONS

The prediction of numerous high-temperature superconducting hydrides is encouraging, which is thanks to the advances in computer hardware coupled with the developments in density functional theory and crystal structure searching tools. However, the quest to identify the most stable structural motifs in generalized convex hulls is undeniably still limited, and other unknown novel stoichiometric and structural motifs may be discovered in the future. Especially, compared with binary systems, the number of possible structures in ternary or multicomponent systems increases exponentially, making their potential energy surfaces very complex, which brings great challenges in theoretical research. As was seen with the crystal structure predictions of the Y–Ca–H system, a simple *Pm*-3*m* YCaH_12_ [85] is the ground state with one unit-cell, while *Fd*-3*m* YCaH_12_ [86], with two unit-cells, is the more energetically favorable ground state and then more complex ground states (such as Y_3_CaH_24_, YCa_3_H_24_ and YCaH_20_) also appear on the ternary phase diagram after considering more stoichiometries [84]. Further increasing the atomic numbers or stoichiometries would greatly expand the phase space of Y–Ca–H compounds, potentially finding more energetically favorable structures, but realizing these would require the performance of energetic and property evaluations on a vast number of structures, raising the computational cost. Meanwhile, the complexity of exploring ternary, quaternary and even polyhydride systems remains a non-negligible challenge to current structure searching simulation methods. Given this complexity, techniques in *ab initio* crystal structure prediction, high-throughput computation, coupled with machine learning (ML) are being applied to find promising candidates and more advanced theoretical computational methods are waiting to be developed [87].

Generally, the theoretical calculations of most hydrogen-rich superconducting materials are based on harmonic approximation. However, ionic quantum fluctuations cannot be neglected, especially for such materials that are rich in light element hydrogen. It is reported that the quantum fluctuations and the consequent anharmonicity significantly affect the dynamic stability, phonon frequencies and superconductivity of H_3_S [88,89] and LaH_10_ [90]. For ternary hydrides, using LaBH_8_ as an example, the quantum expansion of the BH_8_ unit significantly affects the phonon energies [91], which is reflected in the overall softening of hydrogen-character phonon modes, thus increasing the dynamic stability critical pressure. Differently, the anharmonic effects significantly suppress the softening of the phonon vibration modes of BaSiH_8_ and SrSiH_8_ [37] and correspondingly lower their dynamic stability critical pressure. Therefore, the influence of quantum anharmonic effects on hydrides is complex, especially for ternary hydrides with diverse structural motifs, which is also a challenge for future theoretical research. Furthermore, similarly to solid hydrogen [92,93], the nuclei of hydrogen in hydrides have appreciable dynamical properties over a broad range of temperatures [94]. The proton quantum dynamics in these materials deserves more in-depth investigation, especially to reveal their potential impact on superconductivity. It is reported that quantum effects drive the Li_2_MgH_16_ to be in a superionic state at >25 K, which coexist with superconductivity using path-integral molecular dynamics simulations [94] in which protons transfer between interstitial voids in the Li_2_Mg sublattice.

A high-throughput sweep of databases or ML models built upon material data has gradually become the fourth research paradigm, which also has been utilized for binary hydrides [95–97]. However, this has not yet been applied to the calculation of ternary or multivariate hydrides, mainly due to the fact that the structure and properties of ternary or multivariate hydrides are more complex and theoretical predictions and experimental data are relatively short. Therefore, the development of ML will undoubtedly accelerate the research on hydride superconductivity.

## CHALLENGE IN EXPERIMENTAL SYNTHESIS AND MEASUREMENT

Similarly to rich theoretical studies, ternary hydrides also have broad research space in experiments, but they are often elusive due to poor controllability and complex synthesis. First, the synthesis of ternary hydrides is challenging due to the wide range of choices for precursors. When preparing pre-polymers for synthesizing polyhydrides, the most convenient approach for metals with similar properties (e.g. La–Y, La–Ce, etc.) is to use the corresponding alloy. However, difficulties arise when there are significant differences in the melting temperatures and volatility of the metals. This makes the preparation of the initial alloy extremely challenging. Additionally, controlling the stoichiometry or the proportion of elements in the prepared doping compound becomes difficult in the initial precursor. As a result, the synthesis paths for hydrides become highly complex and diverse. Second, different high-temperature and high-pressure synthesis paths directly point to different products. Laser heating of samples to produce mixtures is a common phenomenon in this field. This could bring about the hysteresis of the superconducting transitions during electrical resistance measurement. Moreover, the obtained hydride often contains impurities with by-product phases (e.g. unsaturated lower hydrides) or poor crystallization. Finally, Anderson's theorem for dirty superconductors and non-stoichiometric superconductors states that, in principle, doping cannot increase the *T*_c_ within the framework of the conventional BCS theory. This explains the lower superconducting transition temperature observed in ternary hydrides such as (La, Y)H_10_ [27], (La, Nd)H_10_ [98], (La, Ce)H_9_ [28,29] and (La, Al)H_10_ [30] compared with their parent binary La–H system. Since doping cannot increase the *T*_c_, the next significant goal is to lower the synthesis pressure to make hydrides suitable for practical applications. The synthesized (La, Ce)H_9_ is indeed a good example of reducing the synthesis pressure through non-stoichiometric composition modulation within a suitable host crystal.

## CONCLUSIONS AND PROSPECTS

**Figure 8.**
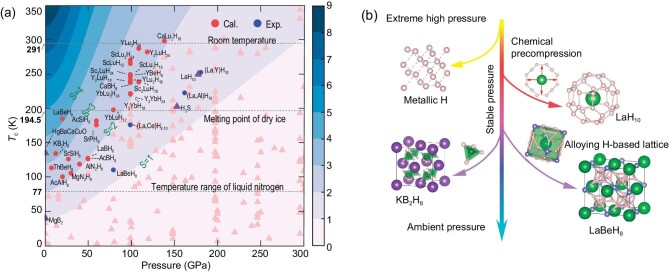
(a) Pressure dependence of *T*_c_ values calculated for superconducting ternary hydrides. The red and blue dots correspond to the theoretical and experimental results of the ternary hydrides, respectively. The pink triangle represents some less prominent results. The purple triangle represents other famous superconductors. The background is shaded according to the figure of merit *S*, which assesses the significance of a particular superconductor ($S = T/\sqrt {{P}^2 + T_{Mg{B}_2}^2} $) [99]. (b) Originated from metallic hydrogen, researchers have made important attempts to lower the stable pressure of superconducting hydrides.

Ternary (or quaternary and higher) hydrides represent the next frontier in the search for superconductors that can host high-temperature superconductivity at increasingly lower pressures. Note that the massive phase space built from all possibilities of combinations of the three elements cannot be thoroughly investigated via current experimental or theoretical techniques. Therefore, precious computational or experimental resources should be focused preferentially on promising systems. Usually, superconductors with excellent properties should be able to maintain a good balance between stable pressure and critical temperature. In order to better evaluate the overall performance of superconductors, the merit *S* is defined [99], which is the result of the critical *T*_c_ value and pressure balance of hydrides. The larger the *S*-value, the better the comprehensive performance of the superconductor. We evaluated the *S*-values of ∼200 different types of ternary hydrides and labeled the theoretically predicted ternary hydrides with *S*-values of >2 and the experimentally synthesized ternary hydrides with *S*-values of >1. Figure 8 clearly shows a very large number of ternary hydrides with higher *S*-values than previous well-known superconductors (e.g. H_3_S [8–10], LaH_10_ [11–16] and MgB_2_ [100], etc.), implying that they have the advantage of hosting high-temperature superconductivity at lower pressures. The ternary hydrides with *S*-values of ≥2 can be divided into two categories. One includes LaBeH_8_ [25], KB_2_H_8_ [38] and their derived isotypic hydrides. The other includes clathrate ternary metal hydrides formed by Lu/Yb elements with full *f*-shells. The *S*-value of LaBeH_8_ is >4, which is the highest value in all ternary systems, followed by KB_2_H_8_, whose *S*-value is >3. Remarkably, most of the ternary systems derived from LaBeH_8_ and KB_2_H_8_ have *S*-values in the range of 2–3, which fully reveals the unique advantages of these two structural motifs. In addition, clathrate alloy hydrides containing Lu/Yb elements also have high *S*-values of >2, such as Y_3_LuH_24_, YLu_3_H_24_, etc. [63].

Coordinating the superior properties of different elements to stabilize more atomic hydrogen is the key for ternary hydrides to exhibit high-temperature and even room-temperature superconductivity. Furthermore, in order to stabilize the superconducting ternary hydrides under moderate pressure or even ambient pressure, future research on superconducting hydrides with outstanding prospects and significance should focus on the following aspects: (i) using appropriate metal elements to precompress the alloyed hydrogen sublattice to stabilize more atomic hydrogen, (ii) ternary or multicomponent alloys, even high-entropy alloys, containing great chemical precompression elements (e.g. Ce, Yb, Lu and Th, etc.) may be a promising method to adjust the comprehensive properties of superconducting hydrides.

Although significant breakthroughs have been made in the search for high-*T*_c_ superconductors in superhydrides under high pressure, many issues, challenges and opportunities remain. A big issue is that hundreds of hydride superconductors have been theoretically predicted, but only a few have been experimentally synthesized. This requires further development of high-pressure experimental techniques and methods, which will undoubtedly promote future research on superconducting hydrides. Theoretical effort will go towards the design of more accessible high-temperature superconducting hydrides. First, it would be helpful to develop a computational method (e.g. kinetic barriers) for evaluating the ease of experimental synthesis. Second, it would be efficient to identify more complex ternary or multivariate superconducting hydrides using ab initio structure searching simulations combining ML. Furthermore, it is necessary to predict the lowest-enthalpy structures and their *T*_c_ values considering ionic quantum fluctuations, although this is a great challenge for ternary hydrides. We believe that with the rapid development of theoretical methods and experimental techniques and their unprecedented synergistic effects, the dream of room-temperature superconductivity will eventually be realized.
